# Komplementäre Pflege in der Onkologie

**DOI:** 10.1007/s00120-023-02217-y

**Published:** 2023-10-25

**Authors:** Sara Kohler, Bernadette Alig

**Affiliations:** Departement Gesundheit, Institut für Pflege, ZHAW – Zürcher Hochschule für angewandte Wissenschaften, Katharina-Sulzer-Platz 9, 8401 Winterthur, Schweiz

Alternativ, komplementär oder integrativ? In Deutschland geben 40 % der Bevölkerung an, eine oder mehrere komplementäre oder alternative Behandlungsmethoden in Anspruch genommen zu haben [3]. Viele Kliniken bieten inzwischen alternative, komplementäre oder integrative Ansätze an. Besonders in der Onkologie sind diese Ansätze sehr gefragt. Für Pflegende eröffnen sich hier neue Möglichkeiten und Herausforderungen.

Um sich dem Thema Alternativ- bzw. Komplementärmedizin zu nähern, bedarf es zunächst einer Begrifflsklärung. Die Definition hat sich in den letzten Jahren immer wieder verändert, jedoch ist diejenige des National Center for Complementary and Integrative Health (NCCIH) eine häufig verwendete: Demnach ist mit Alternativmedizin die Anwendung einer Therapieform, welche nicht der konventionellen Therapie entspricht, als Ersatz gemeint [5]. Komplementärmedizin meint den gemeinsamen Einsatz von Therapien aus dem komplementären Behandlungsspektrum und der konventionellen Therapie [5]. Die Integrativmedizin bettet die komplementären Therapien in das Behandlungsschema ein und stimmt alle Maßnahmen und Therapien aufeinander und auf den Patienten ab (ebd.). Sie kombiniert also konventionelle Methoden mit denen der komplementären Medizin [8].

Witt et al. definieren die integrative Onkologie als patientenzentriertes, evidenzinformiertes Gebiet der Krebstherapie, welches Mind-body-Verfahren, natürliche Produkte und/oder Lebensstiländerungen aus unterschiedlichen Traditionen begleitend zu den konventionellen Krebstherapien einsetzt. Ziel der Integrativen Onkologie ist es, Gesundheit, Lebensqualität und klinische Outcomes über den Behandlungsverlauf hinweg zu optimieren und Menschen zu befähigen, Krebs vorzubeugen und zu aktiven Teilnehmern vor und während der Krebsbehandlung, sowie über diese hinaus, zu werden [9]. Hieraus lassen sich drei zentrale Herausforderungen für Pflegefachpersonen ableiten, die sich mit komplementärer und alternativer Medizin (KAM) befassen möchten:

- das Wissen über Konzepte und Modelle zu KAM,
- das Kennen verschiedener Behandlungsansätze sowie einer Kriterienliste zur Auswahl von Anbietern,
- das Aufzeigen pflegerischer Aufgaben- und Anwendungsbereiche.

## Konzepte und Modelle im Vergleich

Die Reflexion der verschiedenen Begriffe macht deutlich, wie wichtig es ist, die Unterschiede zwischen ihnen zu verstehen, um häufiger Kritik an KAM im Zusammenhang mit Krebstherapien differenziert begegnen zu können. So ist beispielsweise im Zusammenhang mit alternativen Behandlungsmethoden bei Krebs immer wieder von erschreckenden Hungerkuren, durch die der Krebs „ausgehungert“ werden soll und an denen die Patienten aufgrund von Nährstoffmangel versterben oder anderen Radikaltherapien, welche die schulmedizinische Behandlung ausgrenzen, zu hören. Solche rein alternativen Ansätze sollten auf keinen Fall mit einem individuell angepassten Behandlungsplan, welcher komplementäre Ansätze integriert, gleichgesetzt werden. Um den integrativen Ansatz näher zu beleuchten, werden im Folgenden das Konzept der Heilung, dass Modell der Salutogenese und der „Mind-Body Medicine“ (MBM) exemplarisch näher beleuchtet.

Das Modell der Salutogenese: Die Salutogenese geht auf den Soziologen Aaron Antonovsky zurück. Der Begriff setzt sich aus salus (lateinisch: Gesundheit) und genesis (griechisch: Ursprung) zusammen und umschreibt damit den Ursprung der Gesundheit [4]. Antonovsky [1] stellt die bisher sich ausschließenden Konzepte von Gesundheit und Krankheit in ein Kontinuum. Grundlage für die Entwicklung seines Modells war die Frage: „Wie entsteht Gesundheit?“ Nach Antonovsky geht es nicht darum, wie gesund oder krank eine Person ist, sondern wie nahe sie dem Zustand gesund oder krank kommt [4]. Stark vereinfacht könnte man sagen: Man stelle sich einen Fluss (des Lebens) vor, in dem man schwimmt. Dieser birgt Strudel und Untiefen, kann aber auch ganz gleichmäßig fließen. Die salutogenetische Sichtweise möchte dem Menschen helfen, im Fluss zu schwimmen. Die Hilfe soll durch verschiedene Ansätze erreicht werden: fördern, erziehen, vorbeugen, schützen und heilen (ebd.). Das zentrale Konzept für Antonovsky ist der Kohärenzsinn. Er beinhaltet die Verstehbarkeit der Zusammenhänge des Lebens, die Handhabbarkeit von Ressourcen, mit denen man das eigene Leben gestalten kann und die Sinnhaftigkeit der gestellten Lebensaufgabe [7]. Der Kohärenzsinn wird als treibende Kraft des Lebens verstanden [4].

„Mind-Body medicine“: Ein möglicher integrativer Ansatz ist die MBM, welche Betroffenen mit verschiedenen Achtsamkeits- und Bewegungsansätzen hilft, die Krebstherapie und deren Nebenwirkungen physisch und psychisch zu verarbeiten. MBM soll die Lebensqualität verbessern sowie den Stressabbau unterstützen. Es werden beispielsweise Yoga, Meditationen, Hypnose, Ernährungsberatung, soziale Unterstützung, Bewegung oder autogenes Training angewandt [2]. Zwischenzeitlich gibt es einige Kliniken im deutschsprachigen Raum, welche ein Mind-body-Konzept ergänzend zur Behandlung anbieten. Am Universitätsklinikum Zürich können Patienten z. B. einen Kurs über 10 Wochen besuchen, der verschiedene Methoden der MBM erläutert und einführt. Zudem werden online Entspannungsübungen zur Verfügung gestellt. Am Evangelischen Klinikum Essen-Mitte ist eine Mind-body-Therapie stationär, teilstationär und für gewisse Diagnosen auch ambulant möglich. Das Angebot im Bereich MBM wird immer breiter und die wissenschaftliche Grundlage verbessert sich stetig. Wissenschaftliche Untersuchungen legen bereits heute nahe, dass ein Zusammenhang besteht zwischen MBM und der Verminderung von Fatigue und der Verbesserung von Lebensqualität [2].

## Verfahren und Kriterien für die Auswahl des Anbieters

**Abb. 1:**
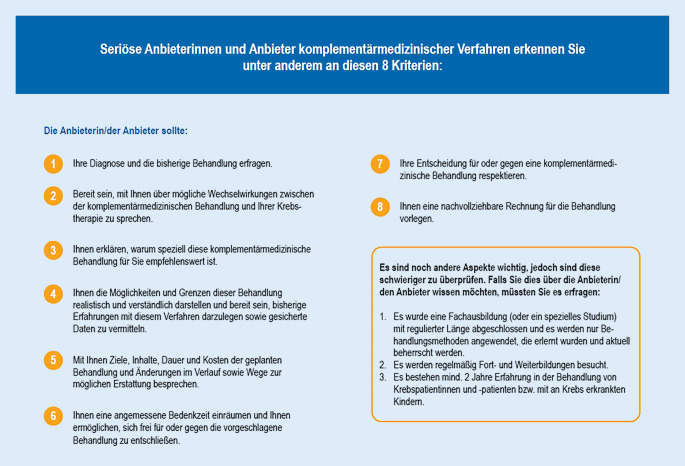
Kriterienliste für seriöse Anbieterinnen und Anbieter komplementärmedizinischer Verfahren. (Aus [6])

Das Angebot an komplementären und alternativen Behandlungsmethoden ist sehr heterogen und selbst innerhalb einer Behandlungsmethode meist verschieden. Die KAM-Methoden können in 5 Kategorien (Tab. 1) eingeteilt werden, welche für eine Übersicht über die Verfahren hilfreich sind [7]. Um Patienten bei der Auswahl seriöser Anbieter zu unterstützen und um ihnen eine gewisse Sicherheit zu bieten, hat das Kompetenznetzwerk Komplementärmedizin in der Onkologie (KOKON) eine Liste mit Kriterien für seriöse Anbieter entwickelt, welche betroffene Personen berücksichtigen sollten (Abb. 1). Darüber hinaus sollte, falls dies möglich ist, auch überprüft werden, ob der Anbieter eine entsprechende Fachausbildung/ein Studium absolviert hat, regelmäßig Fort- und Weiterbildungen besucht und mindestens zwei Jahre Erfahrung in der Behandlung von Krebspatienten hat.

**Tab. 1:** Einteilung von Komplementär- und Alternativmedizin gemäß dem National Center for Complementary and Integrative Health (NCCIH). (Ergänzt nach Schläppi und Kaiser, 2011 [7, S. 189])

Ganzheitliche Medizinische Systeme	Psyche-Körper-Interventionen („mind body“)	Biologische Therapien	Körpertherapien („body based“)	Energetische Therapien
Anthroposophische Medizin (inkl. Misteltherapie), Ayurveda, Homöopathie, traditionelle chinesische Medizin (inkl. Akkupunktur)	Autogenes Training Hypnose, Kunsttherapie, Simonton, Sophrologie	Avemar (Weizenkeimextr.), Curcumin (Gelbwurz), Haifischknorpel, Latrille (Amygladin), Lektin-normierte Misteltherapie, Spurenelemente, Phytotherapie, Ukrain (Schöllkrautextrakt), Vitamine	Chiropraxis, Massage, Osteopathie	„Polarity“, Qigong, „therapeutic touch“

## Aufgaben- und Anwendungsbereiche für Pflegende

Die Gedanken der Salutogenese können Pflegenden bei der Betreuung ihrer Patienten leiten, beispielsweise in dem sie mit der Förderung der personeneigenen Ressourcen einen Beitrag zur Steigerung des Kohärenzsinnes leisten. Eine andere Form pflegerisch-therapeutischer Maßnahmen könnte die Anleitung zur Anwendung von Aromaölen oder äußeren Anwendungen sein. Leidet ein Patient allabendlich unter Einschlafproblemen, kann das Einschlafen beispielsweise durch regelmäßiges Anwenden einer Auflage, welche mit einer Salbe aus Gold, Lavendel und Rose bestrichen wird, eine neue Routine erhalten. Bei dieser Anwendung wird eine Salbe auf ein Baumwoll- oder Leinentuch aufgetragen. Das Tuch kann anschließend durch eine Wärmflasche leicht angewärmt und auf das Herz aufgelegt werden. Die ätherischen Öle wirken durch ihre Nähe zur Nase auf den Geruchssinn und die Wärme strahlt ein wohliges Gefühl aus.

## Patientenschulungen für mehr Selbstmanagement

Neben den bisher beschriebenen pflegerisch-therapeutischen Maßnahmen ist die Schulung und Beratung von Patienten ein weiterer Aufgabenbereich von Pflegefachpersonen. Pflegende in der Praxis werden vor allem Fragen zu Neben- und Wechselwirkungen komplementärer Behandlungsmethoden sowie dem Evidenz- bzw. Empfehlungsgrad begegnen. Gerade Phytotherapeutika haben irrtümlicherweise oft den Ruf, nicht schädlich zu sein oder keine Nebenwirkungen zu haben. Patienten verzichten deshalb möglicherweise darauf, ihrem behandelnden Arzt die Einnahme von Nahrungsergänzungsmitteln oder Phytotherapeutika mitzuteilen. Dies kann gefährliche Folgen haben. Hier ist es die Aufgabe der Pflegenden, eine vertrauensvolle Beziehung zu den Patienten aufzubauen und sie zu bestärken, alle eingenommenen Mittel zu nennen. Ein häufig eingenommenes Präparat im Winter ist beispielsweise das Johanniskraut, welches die Lichtsensibilität erhöht und bei einer Strahlentherapie daher kontraindiziert ist. Auch eine Aufklärung über potenzielle Gefahren oder zu erwartende Nebenwirkungen kann nützlich sein. Die Rotfärbung der Einstichstelle nach einer subkutanen Mistelinjektion beispielsweise gehört zu den erwünschten Nebenwirkungen. Die Einstichstelle kann einen roten Kreis im Umfang eines 2‑€-Stückes aufweisen, der in der Regel innerhalb von 24 h verschwindet. Eine Reaktion wäre allerdings notwendig, wenn die Rötung deutlich größer ist. Die nächste Applikation der subkutanen Mistel sollte dann verschoben werden.

An diesen Beispielen wird deutlich, dass Fachwissen sowie Beratungskompetenz für die Anwendung komplementärer Verfahren erforderlich ist. Um Patienten bestmöglich beraten zu können, ist es hilfreich, den Stand der Forschung zu den verschiedenen Methoden zu kennen. Hier begegnen Pflegende einer weiteren Herausforderung. Bisher gibt es nur wenige Verfahren, die einen soliden Evidenzgrad aufweisen; viele Anwendungen wie die äußeren Anwendungen nach Wegman/Hauschka beruhen jedoch auf breit abgestütztem Expertenwissen und langjähriger Erfahrung. Es gibt inzwischen einige Sammlungen, welche es Interessierten erleichtern, sich einen Überblick über den Forschungsstand zu verschaffen. Die Beratung von Patienten zu komplementärmedizinischen Interventionen erfordert also, analog zur Beratung zu klassischen Behandlungsmethoden, einen hohen Grad an Expertenwissen.

## Weiterbildung für Pflegende

Der Verein „Integrative Kliniken Schweiz“, in dem sich ein Interessenverbund an Kliniken, die KAM anwenden, zusammengeschlossen und Qualitätskriterien definiert hat, empfiehlt eine Weiterbildung von mindestens 150 h, um eine qualitativ gute medizinische und pflegerische Behandlung zu gewährleisten. Bisher gibt es im deutschsprachigen Raum kaum Weiterbildungen, welche den genannten Kriterien entsprechen. Um sich in einem der Bereiche spezifisch zu vertiefen und eine breite Anwendungskompetenz zu erlangen, braucht es konkrete Fachweiterbildungen im gewählten Themenfeld. Pflegende erleben im Kontext der KAM verschiedene Herausforderungen, von denen einige nur durch intensive Weiterbildung oder Vertiefung bewältigt werden, anderen aber durch Achtsamkeit, Gesprächsbereitschaft und Beziehungsgestaltung begegnet werden kann. Der erste Schritt ist stets das Interesse am Themengebiet und die kritische Auseinandersetzung mit Vor- und Nachteilen sowie Vorurteilen (auch eigenen!), um den betreuten Patienten die bestmögliche Versorgung zu ermöglichen.

## Fazit

Die komplementäre und alternative Medizin (KAM) stellt Pflegende vor Herausforderungen: Sie benötigen fachspezifisches Wissen über Konzepte und Modelle zu KAM, sollten Behandlungsansätze und einfache pflegerisch-therapeutische Anwendungen ebenso wie Neben- und Wechselwirkungen kennen und beratende Gespräche führen. All dies findet in der Regel im „normalen“ Arbeitskontext statt. KAM kann eine hilfreiche und effektive Möglichkeit sein, Patienten zu unterstützen und Symptome bei Krebs zu lindern.
